# Tremoroton, a new free online platform for tremor analysis

**DOI:** 10.1016/j.cnp.2019.11.004

**Published:** 2019-12-23

**Authors:** Felipe Vial, Patrick McGurrin, Thomas Osterholt, Debra Ehrlich, Dietrich Haubenberger, Mark Hallett

**Affiliations:** a Human Motor Control Section, National Institute of Neurological Disorders and Stroke, National Institutes of Health, Bethesda, MD, USA; b Facultad de Medicina Clínica Alemana Universidad del Desarrollo, Santiago, Chile; c Parkinson’s Disease Clinic, Office of the Clinical Director, National Institute of Neurological Disorders and Stroke, National Institutes of Health, Bethesda, MD, USA; d Office of the Clinical Director, National Institute of Neurological Disorders and Stroke, National Institutes of Health, Bethesda, MD, USA

**Keywords:** Tremor, Electrophysiology, Software

## Abstract

**Objective:**

The electrophysiological classification of tremors can be a key element in the diagnosis and can facilitate treatment of a patient with tremor; however, the ability to conduct electrophysiological studies of tremor is not widely available. The purpose of this study was to develop and validate a free online platform for tremor analysis.

**Methods:**

An online platform for tremor analysis was developed using “R” language; called “Tremoroton”. For validation, we compared the frequency estimation of the tremor obtained with Tremoroton compared with a commercially available software in a cohort of 20 patients (10 with essential tremor and 10 with Parkinson diagnosis), comparing the activity recorded on the accelerometer, extensor carpi radialis and flexor carpi radialis EMG. An intraclass correlation coefficient was used for the comparison.

**Results:**

The final version of tremoroton is now online. It allows reading up to 6 channels, and will do time, frequency, time-frequency analysis and calculate coherence. We demonstrated a high correlation in frequency measurements (0.97 (0.945–0.984, 95% IC) for the accelerometers, 0.98 (0.977–0.994, 95% IC) for the extensor carpi radialis EMG, and 0.99 (0.987–0.997, 95% IC) for the flexor carpi radialis EMG) when compared to a commercial software.

**Conclusion:**

We were able to develop and validate a free online platform for tremor analysis.

**Significance:**

Making this tool available should help expanding tremor analysis techniques from research to the clinical setting.

## Highlights

• The electrophysiological characterization of tremor is a useful technique.
• There has not been much software available for tremor analysis.
• We developed a free online platform for tremor analysis.

## 1 Introduction

The important classification of tremor in 2018 stressed the need for good phenomenological characterization of tremors and suggested electrophysiology as a useful tool to aid in tremor characterization (Bhatia et al., 2018). In fact, the electrophysiological assessment of tremor can be very useful in differentiating between conditions such as essential tremor, enhanced physiological tremor, functional (psychogenic) tremor and is fundamental for the diagnosis of orthostatic tremor (Vial et al., 2019, Schwingenschuh and Deuschl, 2016, McManis and Sharbrough, 1993).

A standard tremor study of the upper extremities requires an amplifier, surface electromyographic (EMG) recording of forearm extensors and flexors plus wrist accelerometry (ACC) on both limbs (total of 6 channels). By recording different conditions such as rest, posture and posture plus loading, and then analyzing the data in time and frequency domains, the different components of the tremor can be extracted (mechanical, mechanical reflex and central component). Also, in the case of a central component, the presence of one or more oscillators can be determined. All of these parameters are very useful to narrow down the differential diagnosis in a patient with tremor (Vial et al., 2019).

The ability to conduct electrophysiological studies for tremor are not widely available. Considering that the hardware needed is standard in most electrophysiology labs, we suspect that the biggest limitation to perform these studies may be due to the lack of available software options.

The purpose of this study was to develop and validate a free online platform for tremor analysis using open source tools.

## 2 Methods

### 2.1 Application development

Software was written in “R” language using RStudio, a free open source integrated development environment (IDE). We call this software “Tremoroton”.

It was designed to receive acquired tremor data as a text file (.txt), which is a universal format to which almost every type of electrophysiological dataset can be transformed. This enables use of the software regardless of which system was used to acquire the data. The platform was developed with the ability to receive data from 6 channels (1 channel for accelerometry and two EMG channels per limb) at any sampling rate.

#### 2.1.1 Data preprocessing

The channels are separately preprocessed in the following manner; the accelerometry (ACC) channels are high pass filtered at 2 Hz and low pass filtered at 30 Hz in order to detect the frequencies of all known organic tremors that range between 3 (cerebellar tremor) and 18 Hz (orthostatic tremor), the EMG channels are high pass filtered at 20 Hz and low pass filtered at 300 Hz to be able to capture most of the frequency content of the EMG signal(Nilsson et al., 1993, Vial et al., 2019). This is done with third order Butterworth filters. The EMG channels are also rectified and smoothed as recommended for frequency analysis in the setting of tremor (Milner-Brown and Stein, 1975).

#### 2.1.2 Frequency analysis

The frequency analysis is performed by a fast Fourier transformation over the full segment of data on each channel (Hallett, 1998).

#### 2.1.3 Magnitude squared coherence

The data is first segmented according to the parameters set by the user. Each segment is then tapered at the beginning and the end with a split cosign wave. Then the magnitude squared coherence is calculated and all the segments are averaged.

The 95% confidence limits and confidence intervals for the coherence are calculated according to the method described by Halliday (Halliday et al., 1995).

#### 2.1.4 Spectrogram

The spectrograms are obtained by calculating the Fourier transform of sliding ∼1 s windows (an approximation of the sampling rate number of points to the next power of 2) with a 50% overlap. The data are displayed in a time by frequency by power spectrogram.

### 2.2 Validation

For the validation, we performed a retrospective analysis of data from 20 patients (10 essential tremor (ET) and 10 Parkinson disease (PD)) in which tremor studies were previously performed. The data used were collected under a local IRB approved protocol for which subjects sign a consent form. The data were recorded with Viking EDX machine (Nicolet Biomedical - Madison, WI). EMG was recorded from bilateral extensor carpi radialis and flexor carpi radialis muscles plus wrist accelerometer for 30 s sampling at 1000 Hz. For the ET cases, we used recordings taken while the patient was holding hands in outstretched posture with both forearms resting on arms of an armchair. For the PD cases, we used the tremor recorded during rest (with forearms resting on the armchair arms and hands hanging over the edge of an armchair). The data from the ACC, extensor carpi radialis EMG (ECR) and flexor carpi radialis EMG (FCR) were analyzed with commercially available tremor analysis software (Lauk et al., 1999) and with Tremoroton. The peak frequencies were extracted for both hands and analyzed with an intraclass correlation coefficient (ICC). A two-way model, single-rater, absolute agreement was used. Based on the 95% confidence interval, values less than 0.5, between 0.5 and 0.75, between 0.75 and 0.9, and greater than 0.90 were used as the ranges for poor, moderate, good, and excellent reliability, respectively(Koo and Li, 2016).

## 3 Results

### 3.1 Online application

The platform was developed and successfully uploaded on a website. It can be accessed on https://electrophysiology.shinyapps.io/Tremoroton/. We have included sample data as supplemental material to try the platform (Supplement 1, 2 and 3), and there is also an example of a typical tremor analysis (Supplement 4).

**Fig. 1:**
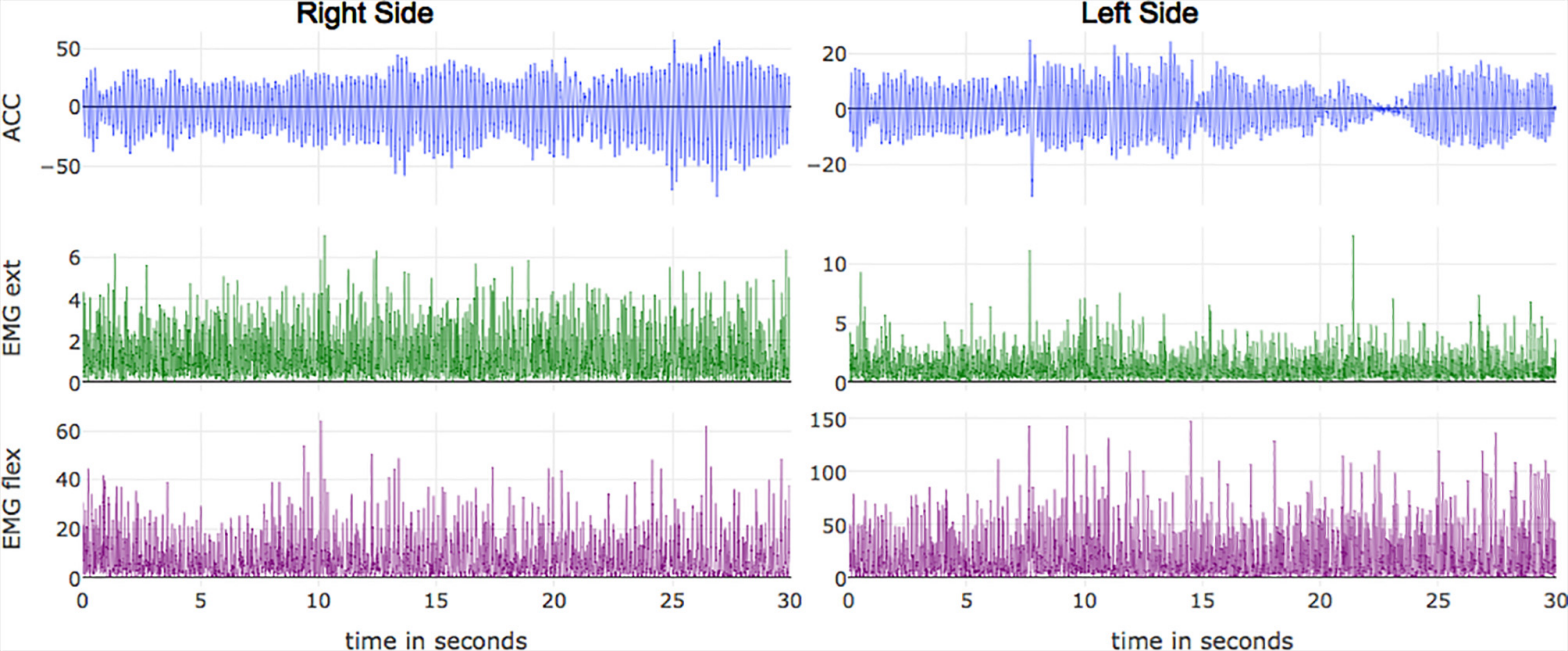
General data visualization: The ACC and EMG channels are extracted from the text file and visualized. Each data channel derived from a particular column in the text file must be specified in the platform. Additionally, the sampling rate must be specified.

**Fig. 2:**
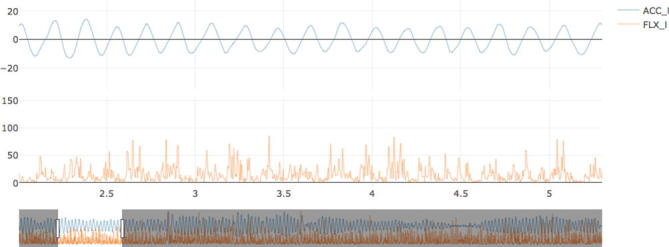
Detailed visualization of two channels: Two channels can be selected for detailed visualization. On the bottom of the two channels there is a scrolling bar that allows detailed visualization of a particular segment of the data.

**Fig. 3:**
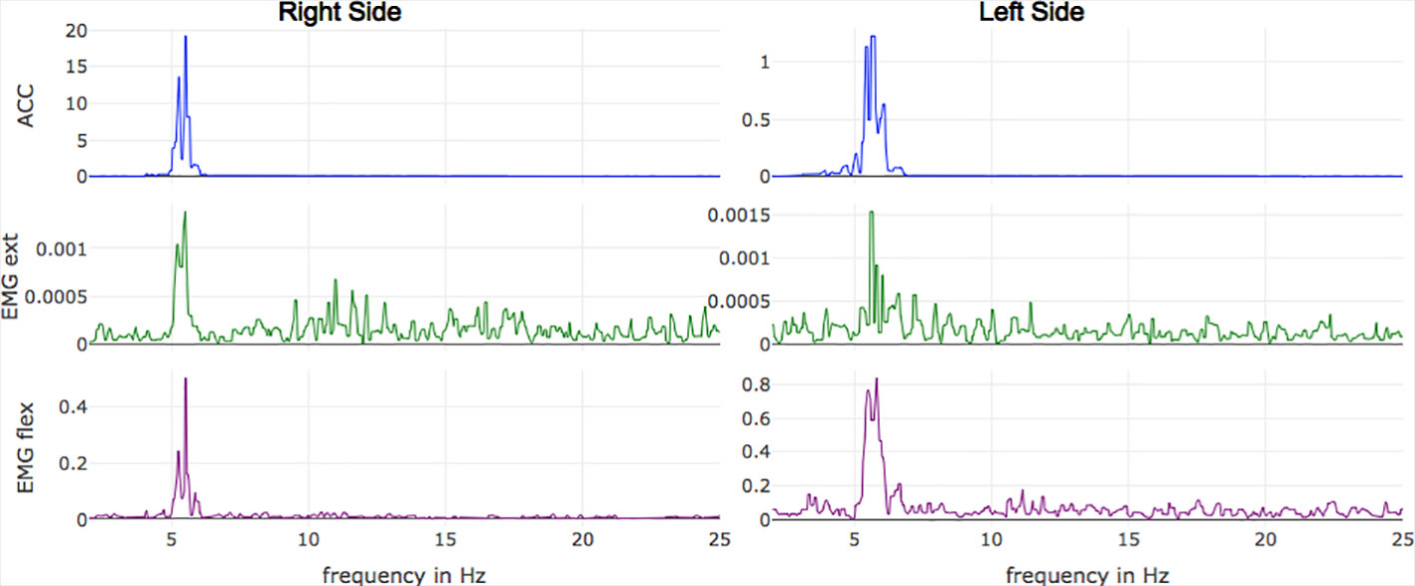
Transformation to the frequency domain: The result of the FFT is presented for each channel. By hovering the cursor over the plot, the specific frequency of the peak can be seen.

After the data is loaded into Tremoroton, it enables visualization of the 6 channels in the time domain using interactive plots (the x and y axis can be changed by clicking and dragging the plot and the value at each point can be visualized by hovering the mouse over the trace) (Fig. 1). It also enables the selection of two specific channels for a more detailed analysis in the time domain (Fig. 2). The frequency domain analysis can also be visualized using interactive plots (Fig. 3).

**Fig. 4:**
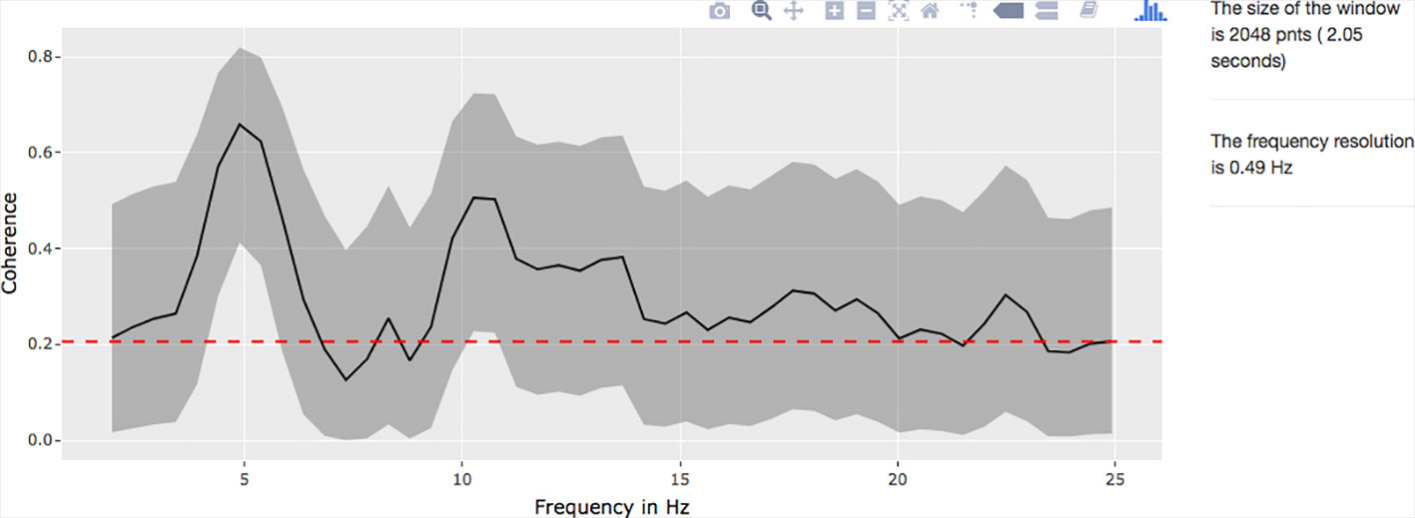
Coherence between two selected channels: Magnitude squared coherence can be calculated between two specific channels. The window size for the coherence analysis must be specified. The 95% confidence limit is demonstrated as a dashed red line and confidence intervals as a grey ribbon. (For interpretation of the references to color in this figure legend, the reader is referred to the web version of this article.)

The results of the magnitude squared coherence can be calculated between two channels and the data can be visualized with 95% confidence intervals (Fig. 4).

**Fig. 5:**
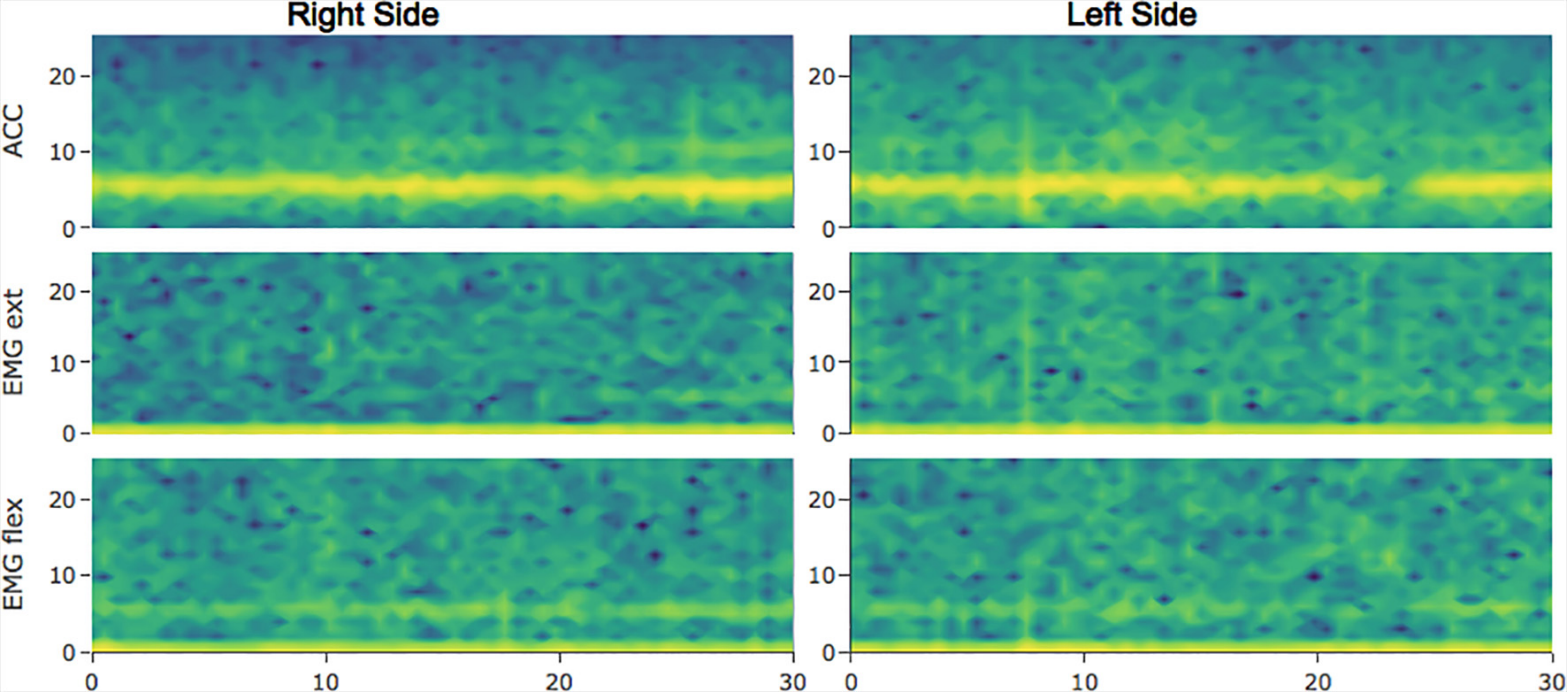
Spectrogram: For each channel, the spectrogram shows the time in the “x” axis, frequency in the “y” axis, and power for each frequency in the “z” axis (expressed as a color scale).

**Fig. 6:**
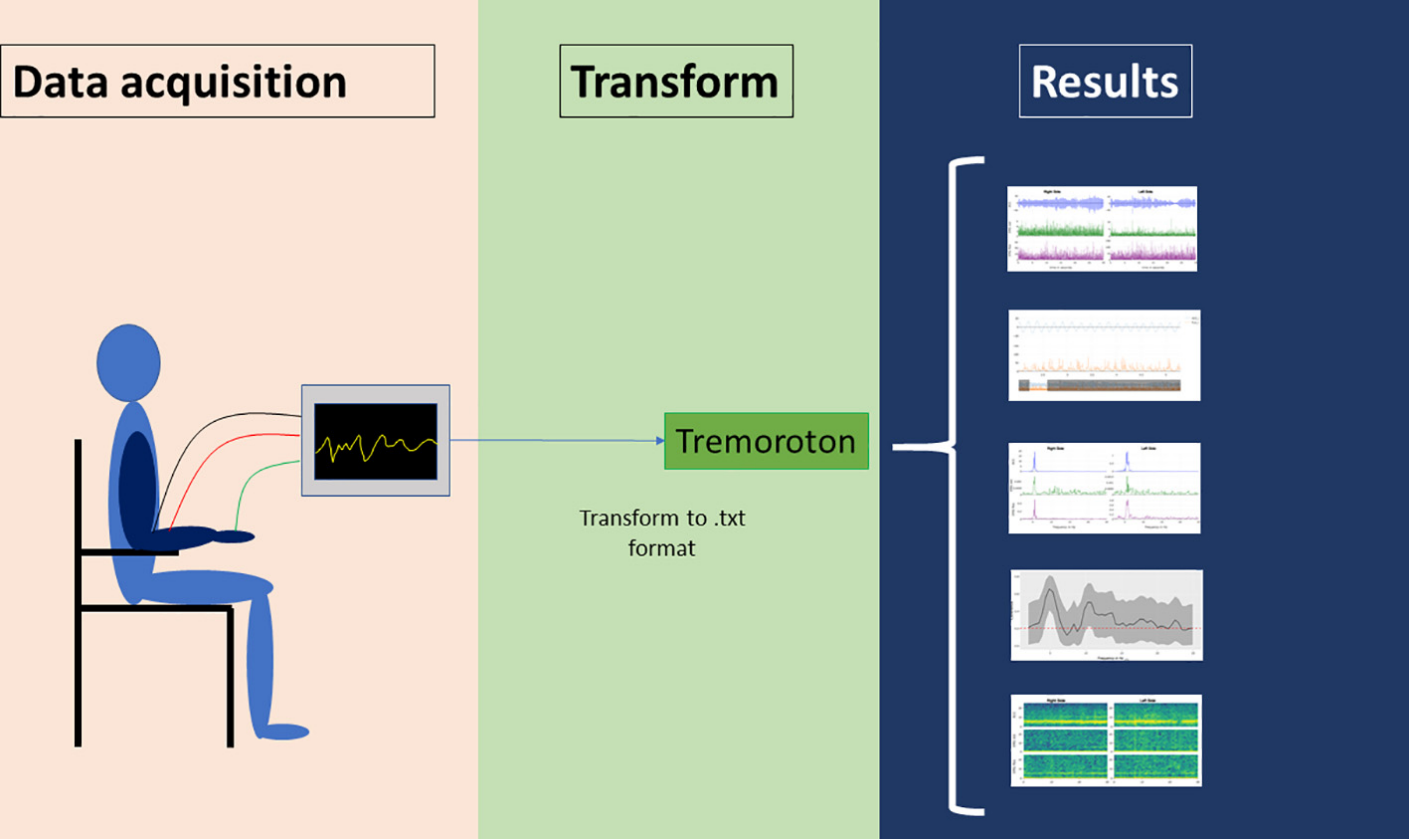
Summary: The data are acquired with the available hardware, then have to be transformed to .txt files, and then it can be processed with Tremoroton platform.

Finally, a spectrogram representation of the data can be produced to visualize the changes in frequency over time (Fig. 5). A summary of the whole process is diagrammed in Fig. 6.

### 3.2 Validation

**Fig. 7:**
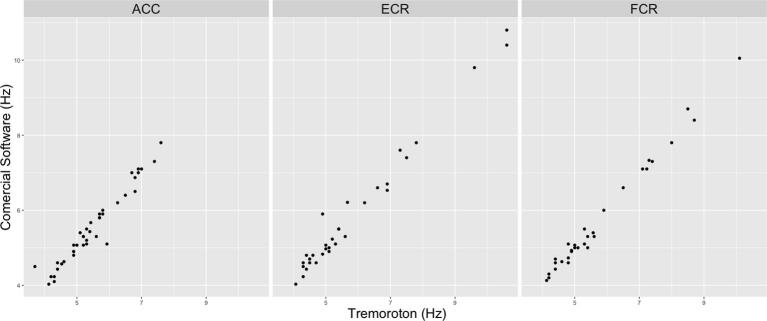
Correlation scatter plot: The plots demonstrate a strong correlation between our new online platform compared to a commercially available software for ACC, ECR and FCR data.

A scatterplot of the frequency correlation between the commercial software and Tremoroton is shown in Fig. 7. The intraclass correlation coefficients were 0.97 (0.945–0.984, 95% IC) for the ACC, 0.98 (0.977–0.994, 95% IC) for the ECR and 0.99 (0.987–0.997, 95% IC) for the FCR. In the 3 cases “p” was less than 0.001.

## 4 Conclusions

We offer a free web-based tool for tremor analysis with excellent reliability compared to an available commercial option, entirely developed with open source tools. We are also releasing the codes to the community, which can be adapted for their own needs.

We hope that the availability of this software will reduce existing barriers to conducting electrophysiological tremor studies.
