# Supplementary material for: Tremoroton, a new free online platform for tremor analysis
Source: Clin Neurophysiol Pract. 2019 Dec 23;5:30–4. doi: 10.1016/j.cnp.2019.11.004 (PMC6961062; doi:10.1016/j.cnp.2019.11.004)
Supplement: Supplementary data 3 [file mmc3.docx]

Information about the dataset:

There are two datasets from the same patient with essential tremor. Data was acquired while the patient was sitting with both hands extended and forearms support by the armrest for 30 seconds (Supplement_1) and then the same posture with 2lb added to each limb (Supplement_2).

The data were sampled at 1000 Hz and the order of the channels are:

1: time

2: right accelerometer

3: right extensors

4: right flexors

5: left accelerometer

6: left extensors

7: left flexors

Instructions:

1.- Download the Supplement_1 file.

2.- Input the channels: The first channel “time” is not needed because time is calculated from the sampling rate. The rest channels need to be put in the corresponding boxes in the “General View” window of the platform (for this data set, is not necessary because the number channel order is already there as default).

3.- Browse the Supplement_1 file.

4.- Following that. it is possible to move to the “Time domain”, “Frequency domain”, “Coherence” or “Spectrogram” window. There is more information about each section in the “Documentation” window.
