# Supplementary material for: Tremoroton, a new free online platform for tremor analysis
Source: Clin Neurophysiol Pract. 2019 Dec 23;5:30–4. doi: 10.1016/j.cnp.2019.11.004 (PMC6961062; doi:10.1016/j.cnp.2019.11.004)

Tremor Study Report

Indication and referral question:

Assessment for a bilateral posture and action tremor.

Recording and data processing:

EMG polygraphy was performed using a setup including 4-channel surface EMG, and 2-channel accelerometry. EMG electrodes were placed in a bipolar montage bilaterally over the wrist extensors (extensor digitorum communis - EDC) and wrist flexors (flexor carpi ulnaris - FCU). Accelerometers (Kistler, sensitivity 20mV/g) were placed at the back of the hands and recorded in the z-axis.

Recording conditions were 30 seconds each and involved posture with hands outstretched and arms supported, posture with hands outstretched and arms supported plus the addition of one pound weight to each hand, posture with hands outstretched and arms supported plus the addition of 1.5 pounds, and posture with hands outstretched and arms supported plus the addition of two pound weight to each hand.

EMG data were analyzed using Tremoroton.

Results:

During posture, there is a 7Hz tremor on the right and around 6Hz tremor on the left hand, both recorded on accelerometer and EMG channels. This bilateral tremor is persistent on its frequency during weight loading. During posture, it is also possible to observe a bilateral 4-5 Hz peak just on the accelerometers channels that shift to approximately 3 Hz with weight loading, without a reproducible EMG correlate.

Interpretation:

The tremor of this patient has a bilateral, non-weight dependent, central component at approximately 6-7 Hz. In addition, there is a weight-dependent 3-5 Hz component compatible with a physiological tremor (peripheral, mechanical component). This is consistent with essential tremor.

Posture

Time Domain

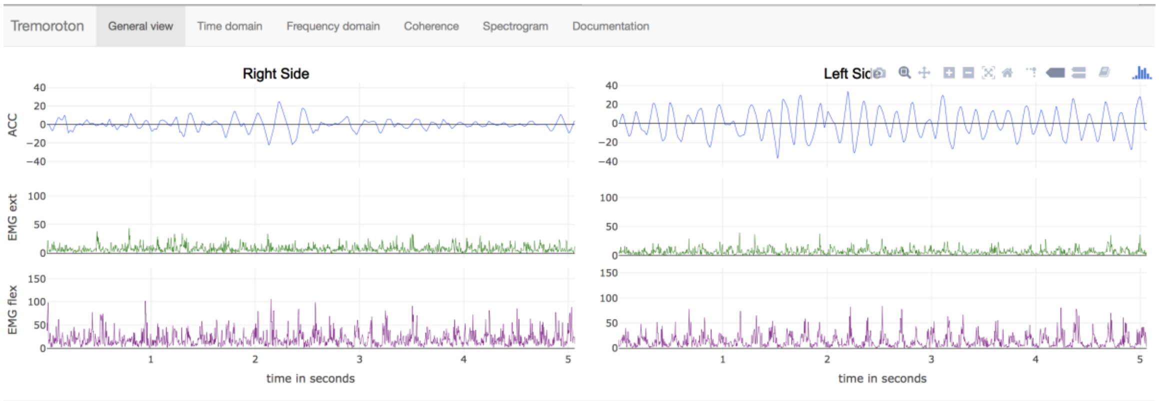

Frequency Domain

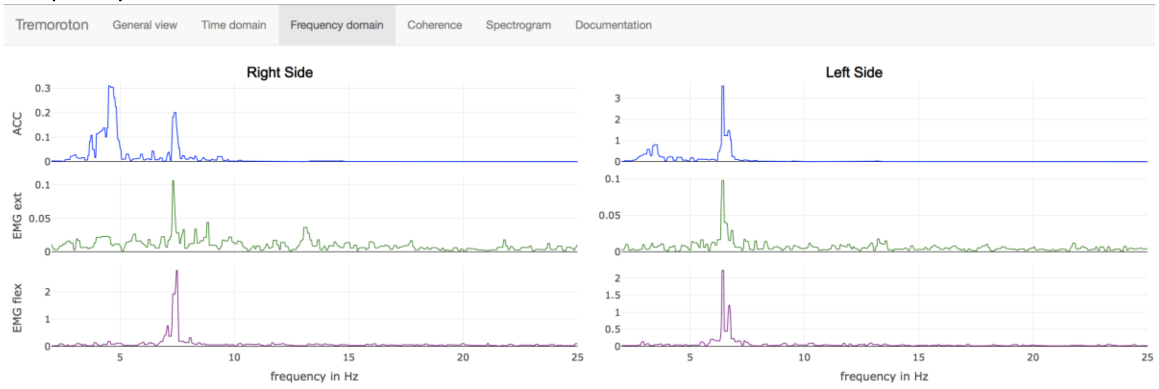

Spectrogram

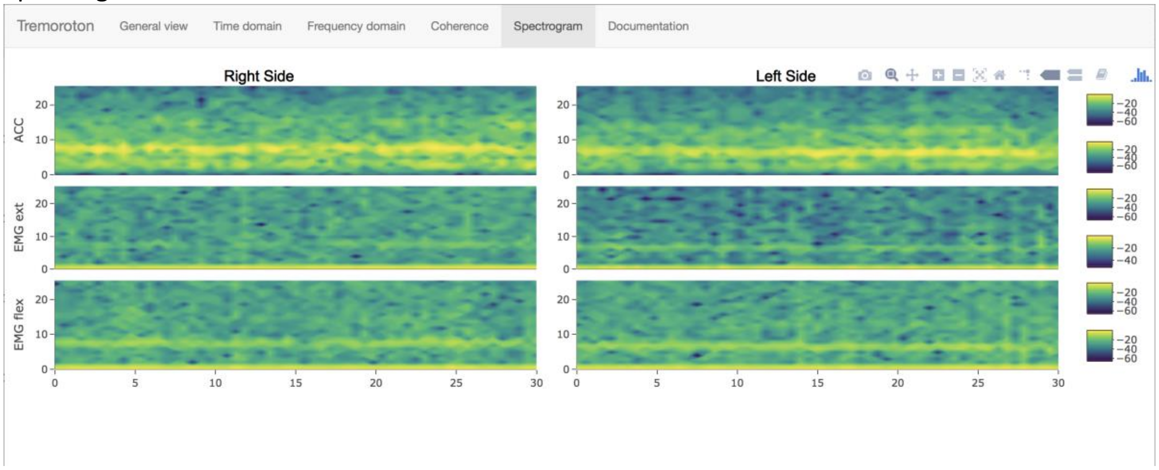

Posture with 1 lb. loading

Time Domain

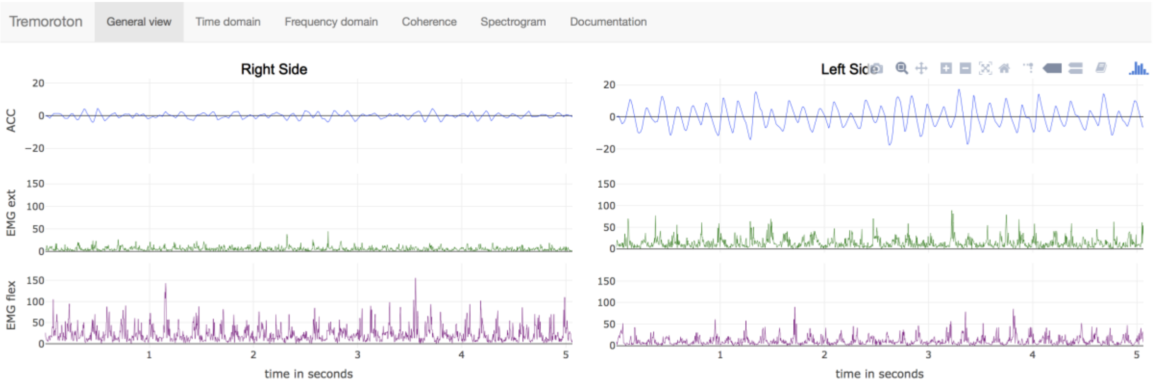

Frequency Domain

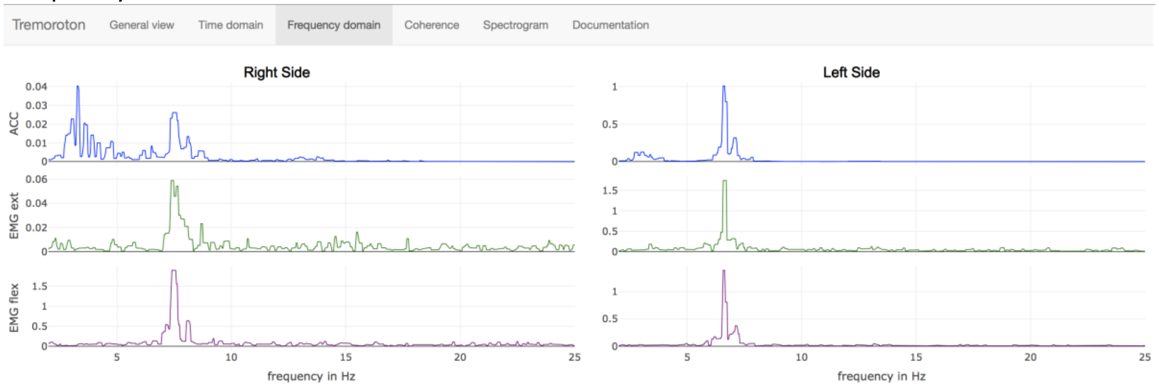

Spectrogram

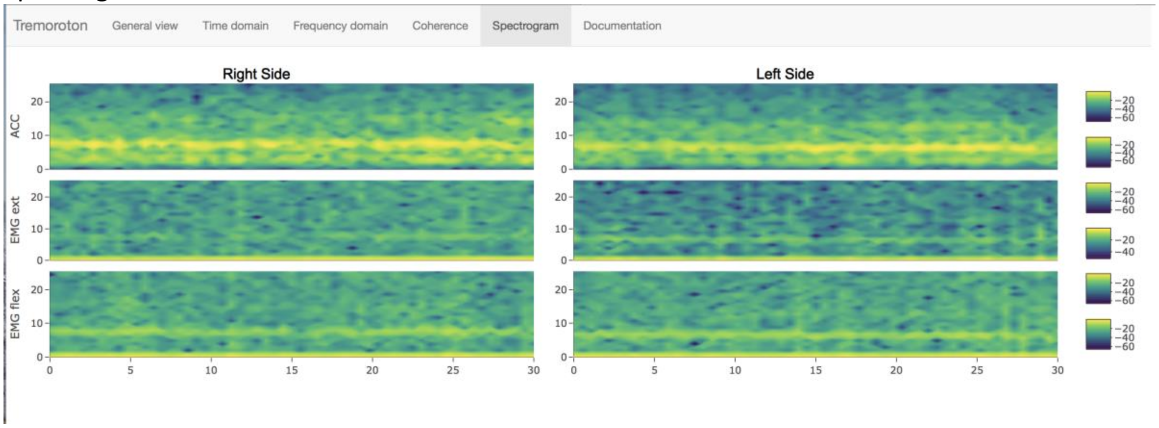

Posture with 1.5 lbs. loading

Time Domain

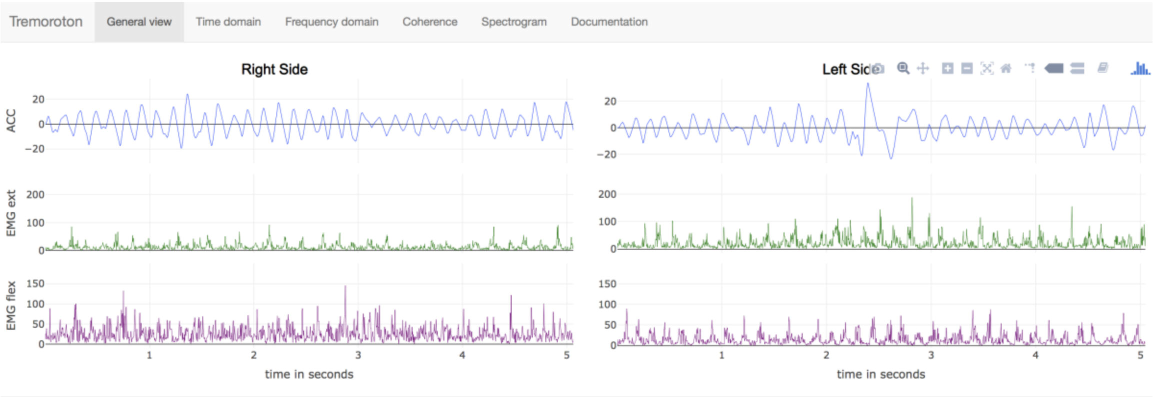

Frequency Domain

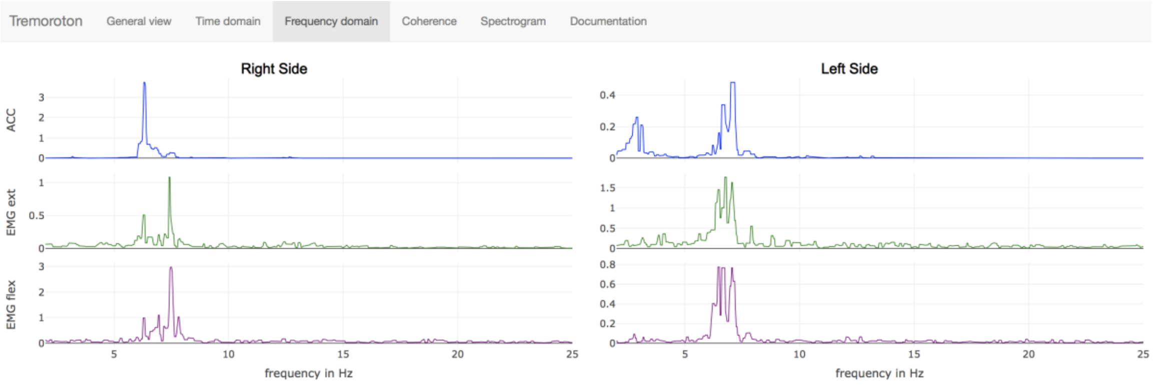

Spectrogram

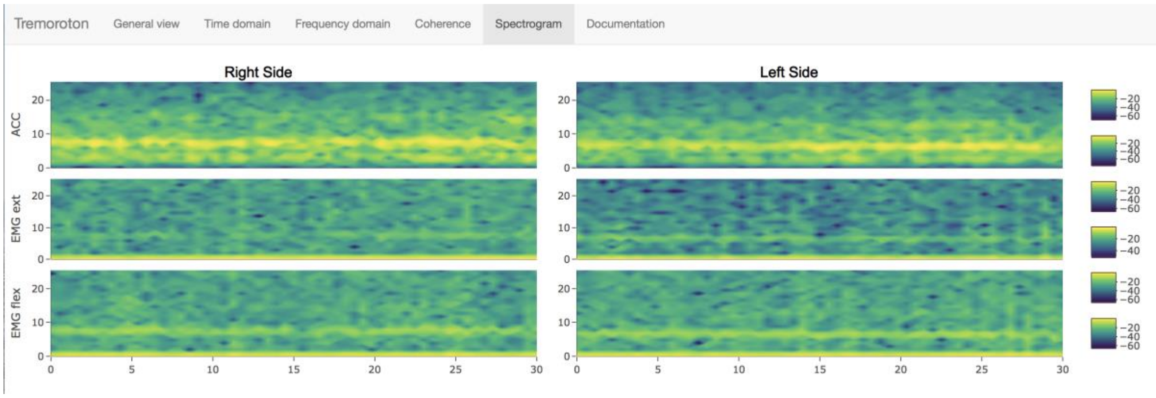

Posture with 2 lbs. loading

Time Domain

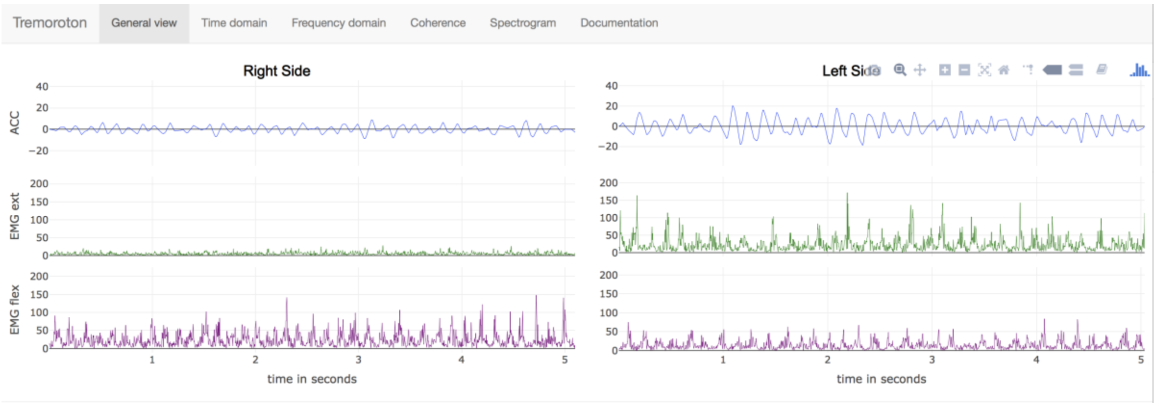

Frequency Domain

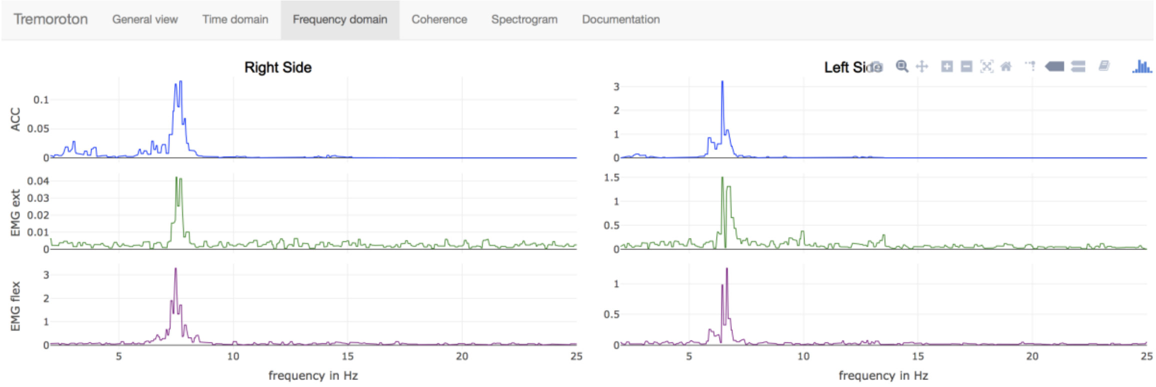

Spectrogram

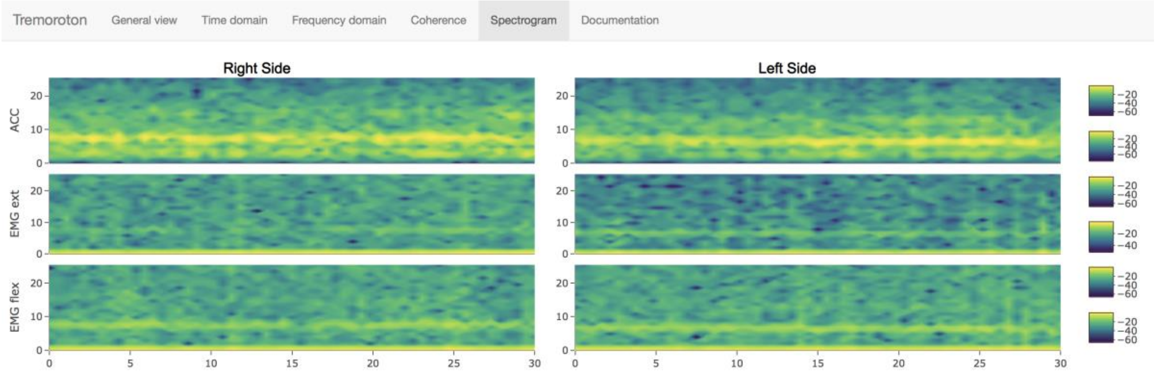

Supplement: Supplementary data 4 [file mmc4.pdf]
